# Potential Role of Garcinol as an Anticancer Agent

**DOI:** 10.1155/2012/647206

**Published:** 2012-06-13

**Authors:** Nadia Saadat, Smiti V. Gupta

**Affiliations:** Department of Nutrition & Food Science, Wayne State University, Detroit, MI 48202, USA

## Abstract

Garcinol, a polyisoprenylated benzophenone, is extracted from the rind of the fruit of *Garcinia indica*, a plant found extensively in tropical regions. Although the fruit has been consumed traditionally over centuries, its biological activities, specifically its anticancer potential is a result of recent scientific investigations. The anticarcinogenic properties of garcinol appear to be moderated via its antioxidative, anti-inflammatory, antiangiogenic, and proapoptotic activities. In addition, garcinol displays effective epigenetic influence by inhibiting histone acetyltransferases (HAT 300) and by possible posttranscriptional modulation by mi RNA profiles involved in carcinogenesis. *In vitro * as well as some *in vivo * studies have shown the potential of this compound against several cancers types including breast, colon, pancreatic, and leukemia. Although this is a promising molecule in terms of its anticancer properties, investigations in relevant animal models, and subsequent human trials are warranted in order to fully appreciate and confirm its chemopreventative and/or therapeutic potential.

## 1. Introduction

The extract from the fruit of *Garcinia indica*, popularly known as* Kokum* or *Mangosteen *has been valued in the Indian subcontinent, Africa, and China for its sweet and sour taste and has traditionally been used as a seasoning, a snack, or steeped in syrup for a refreshing drink. In addition, it has been recommended by the Ayurvedic system of medicine for treatment of ailments such as heat strokes, infections, and edema [1].

The major chemical constituents of the fruit extract include citric acid, hydroxycitric acid (HCA), hydroxycitric acid lactone, and oxalic acid in addition to the benzophenone derivatives, garcinol, and its isomer isogarcinol [1]. Of these, HCA has shown hypocholesterolemic and antiobesity activity, while the benzophenone derivatives have been associated more closely with antioxidant behavior. In this paper, we will concentrate on the biological properties of garcinol with a specific focus on its anticarcinogenic capabilities.  Figure 2 gives an overview of the mechanistic insight into the anticancer activity of garcinol.

## 2. Chemical Properties of Garcinol

Garcinol (C_38_H_50_O_6_; molecular weight 602), a yellow crystalline compound, with a melting point of 132°C is structurally similar to curcumin, an established antioxidant, antiaging, and anticarcinogenic agent, by virtue of containing both phenolic hydroxyl groups and the *β*-diketone moiety [2].

**Figure 1:**
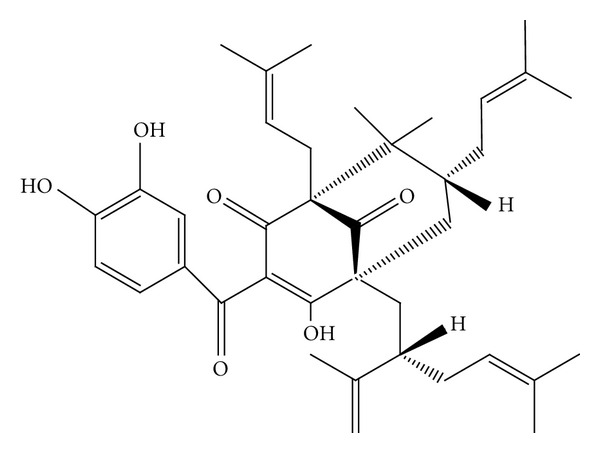
Chemical structure of Garcinol [15].

**Figure 2:**
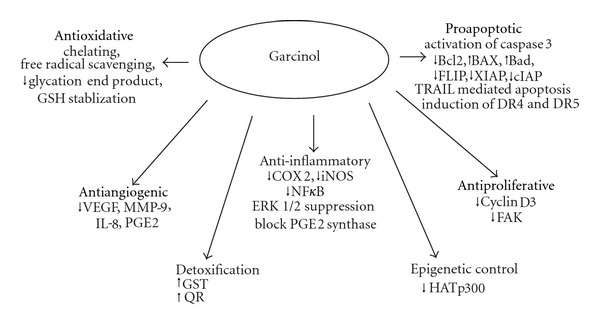
Anticancer activity of Garcinol: mechanistic targets.

The chemical structure of garcinol, also known as Camboginol (Figure 1), was elucidated by Rao and coworkers in 1980 using proton NMR and IR spectroscopy where they determined the location of a terminal alkene as well as the presence of a *β*-diketone from the compound's ability to enolize [2]. The terminal alkene contained in an unsaturated isoproprenyl substituent of the molecule can undergo cyclization under acidic conditions to produce isogracinol [3]. The latter, oxidized isomer of garcinol has also been associated with antitumorigenic, antiobesity, antiulcer, antibacterial, antiviral, and anti-inflammatory properties [4]. The chemical structure of garcinol is similar to that of a series of naturally occurring phytochemicals such as the chalcones, oglogifolins, and Guttiferones [5, 6]. Liquid chromatography-tandem mass spectrometry method to rapidly and sensitively detect the presence and quantify the amount of garcinol present in a plant extract has now been developed, paving the way to a rapid source of Garcinol and hence a higher propensity of scientific investigations in this area [7].

The chemistry of garcinol and its synthetic chalcone analogs has been detailed in an earlier review [1]. In brief, as compared to the native chalcones fluorinated 2 hydroxychalcones have been reported to display increased antioxidative potential and bioavailability, which was exhibited effectively in pancreatic (BXPC 3) and breast cancer (BT-20) cell lines [8].

## 3. Antioxidative Properties of Garcinol

Oxidation and inflammation are considered to be underlying common causes of many chronic diseases including atherosclerosis, Alzheimer's' disease, cancer as well as the normal process of aging. Specifically, oxidative stress, the imbalance between a system's oxidative and the antioxidative pathways, in favor of the former, is brought on primarily by free radical species, resulting in cellular damage [9]. Antioxidants, by definition, inhibit oxidation by either inhibiting pro-oxidants, thereby preventing the formation of free radical species or by retarding the rate of reaction of oxidative species with their biological targets, thereby slowing down the free radical chain propagation [9]. Since the free radical chain, if allowed to propagate, can follow multiple pathways leading to a vast number of end products, measurement of oxidative stress or a substance's antioxidative ability has always been considered to be precise, relative to a specified biological condition or status, at best. Owing to the large number of molecules affected by free radicals, leading to a varying population of end products, a sizable array of independent assays have been put forward for the measurement of oxidative stress or an agent's antioxidative capacity.

The antioxidative ability of garcinol has been investigated in* in vitro* and *in vivo* model systems [10]. Using a hypoxanthine/xanthine oxidase system, garcinol was shown to retard superoxide anion to nearly the same amount as DL-alpha-tocopherol, an established anti-oxidant, while its ability to quell hydroxyl radicals in the Fenton reaction system was even better than that of alpha-tocopherol. In addition, the authors also explored the antioxidative power of garcinol *in vivo* using an indometacin induced rat model for acute ulceration. Oral administration of garcinol prevented acute ulceration in these rats, suggesting its potential as an antiulcer drug [10]. In another study, glutathione levels, known to be depleted due to oxidative stress in the erythrocytes of streptozotocin-induced type-2 diabetic rats, were effectively restored by oral administration of an aqueous extract of the fruit of *Garcinia indica *[11]. This could be attributed to the anti-oxidant potential of garcinol in the fruit extract. The protective role of garcinol in neuronal survival and differentiation was investigated in cultured cortical progenitor cells. [12]. In another study, antioxidative and neuroprotective properties of garcinol in rat cortical neuron cultures was observed. This was suggested to occur via prevention of nitric oxide NO accumulation in lipopolysaccharide treated astrocytes [13].

The examples above help to establish the antioxidative effect of garcinol *in vitro* and *in vivo* model systems. In terms of its structure, the phenolic hydroxyl groups coupled with the *β*-diketone moiety in garcinol may, via formation of resonance stabilized intermediates, help to prevent the free radical species from propagating and thereby limiting further oxidative-stress-related damage down the road.

## 4. Anticancer Properties of Garcinol

Cancer, the uncontrolled growth and spread of abnormal cells, may be initiated due to multiple factors including exposure to carcinogens, repeated genetic damage, by oxidative stress, chronic inflammation, or hormonal imbalance. Once initiated, a cascade of reactions ensues, making it difficult to specify molecular targets for therapeutic advancements. As such, most chemotherapeutic treatments suffer from adverse toxic reactions leading to acute and delayed nausea, mouth ulcerations, fatigue, nerve damage, blood clots, anemia, and mild impairments [14]. Thus, it is important to develop effective preventative and/or therapeutic approaches, which could potentially be both effective against cancer cell growth and relatively nontoxic. Recent evidence supports that nonnutritive components in diet have therapeutic benefits attributable to their pleiotropic effects including downregulation of survival signaling and simultaneous activation of multiple death pathways in cancer cells.

A number of recent studies have examined the potential of garcinol, a non-nutritive dietary component, against different cancer types (Table 1). In the process, a number of possibilities that explain the underlying mechanism for the chemopreventative and/or therapeutic performance of garcinol have also arisen. These are summarized below.

### 4.1. Garcinol: Effect on Inflammatory Pathways

The effect of dietary garcinol on the development of azoxymethane- (AOM-) induced colonic aberrant crypt foci (ACF), precursors for colon cancer, was investigated in male F344 rats [15]. Ingestion of 0.01% and 0.05% dietary garcinol in rat models significantly reduced the formation of ACF in a dose-dependent manner, thus suggesting suppression in cancer development [15]. No adverse effects were reported with diets containing up to 0.05% by weight of garcinol [15]. Additionally, garcinol consumption provided the added benefit of increased liver glutathione S-transferase (GST) and quinone reductase (QR) levels, both detoxifying enzymes associated with cancer suppression [15, 33].

Since carcinogenesis is often accompanied with increase in oxidative stress, the authors also probed oxidative and inflammatory pathways and investigated the response on O2-, Nitric Oxide (NO), iNOS, and COX2 activity upon treatment with garcinol *in vitro*. Garcinol inhibited O2-, Nitric Oxide (NO), iNOS, and COX2 at a slightly greater level than the green tea polyphenol, epigallocatechin gallate (EGCG), an established plant antioxidant, in the cell lines evaluated [15]. Taking the two together, it is possible that the anticolon tumorigenesis activity of garcinol may in part be due to modulation of iNOS and COX2 activities, although mechanistic details need further elucidation [15].

The COX-2 expression in tongue lesions induced with 4-nitroquinoline 1-oxide (4-NQO) in male F344 rats was also decreased on feeding garcinol to these animals [32]. In this study, dietary garcinol was administered either during the period of exposure to the carcinogen or after the exposure to carcinogen was complete. The animals fed the garcinol-containing diet had significantly decreased the incidence and multiplicity of 4-NQO-induced tongue neoplasms and/or preneoplasms as compared to the control diet under both conditions. This shows the potential of garcinol both as a preventative and therapeutic agent against tongue cancer. In addition, the authors did not observe adverse effects with respect to survival rate, or any histological changes in the liver or kidney that could be related to toxicity.

In another study, the mechanisms by which garcinol modulates arachidonic acid metabolism and NO synthesis in lipopolysaccharide- (LPS-) stimulated RAW264.7 murine macrophages and in intestinal cell lines were examined [34]. Treatment with garcinol decreased the release of arachidonic acid and its metabolites in the cell lines tested. Results indicate that the suppression of arachidonic acid on treatment with garcinol after the activation of LPS-stimulated macrophages may occur via inhibition of cPLA_2 _and ERK 1/2, key factors in the release of arachidonic acid from membrane phospholipids [34]. Conversely, garcinol treatment prior to LPS-stimulation may lead to inhibition of the toll-like receptors upstream of ERK 1/2 [34]. Additionally, downregulation of NF-*κ*B and COX-2 expressions, both involved in the inflammatory response were also observed [34].

Koeberle and coworkers showed that garcinol interferes with 5-lipoxygenase and microsomal prostaglandin PGE2 synthase enzyme activity, both of which play crucial roles in inflammation [29]. Further, garcinol inhibited synthesis of PGE2 and 5-lipoxygenase in human neutrophils and interleukin-stimulated human lung carcinoma cells [29]. The authors established that 5-lipoxygenase and certain prostanoids are possible targets of garcinol, and may be of potential pharmacological interest [29].

Prostaglandin E2 (PGE2) is produced by the action Cox2 enzyme. It has been documented that aggressiveness of pancreatic cancer increases with increase in the level of PGE2 [35]. Targeting PGE2 can decrease inflammation and proliferation and can reduce metastasis of pancreatic cancer through various factors like MMP-2 and MMP-9 [36, 37]. In our laboratory, we have observed significant reduction in PGE2 expression levels in Cox 2-containing pancreatic cancer cell line BXPC3 with treatment with garcinol.

Taken together, these studies demonstrate that inhibition of inflammatory pathways via multiple but related targets may play a pivotal role in garcinol's response as an anti-inflammatory and anticarcinogenic agent.

### 4.2. Garcinol: Effect on Apoptotic Pathways

Another approach for halting cancerous growth involves targeting apoptosis, programmed cell death of rapidly dividing cancer cells. Garcinol exhibited greater induction of apoptosis than curcumin in human leukemia HL-60 cells [28]. This was mediated by release of cytochrome c into the cytoplasm of the cell from the mitochondria allowing for the activation of caspase-3. Apoptosis was also accompanied with downregulation of Bcl-2, an antiapoptotic protein and significant upregulation of Bad and Bax, established proapoptotic proteins. The results suggest that apoptosis affected by garcinol is initiated by the release of cytochrome c into the cytosol, followed by procaspase-9 processing, activation of caspase-3 and caspase-2, degradation of PARP, and DNA fragmentation [26–28].

Interestingly, when Hong and coworkers investigated the effect of garcinol on colon cancer and normal immortalized intestinal cell line, garcinol showed a much higher potency of inhibition against the colon cancer cells as compared to the normal cells. Greater growth inhibition was accompanied by increase in Caspase 3 activity, indicative of an apoptotic pathway [30]. This differential response in activity against cancer cells versus normal cells was also observed in breast cells [17, 20]. However, at exceptionally low doses (<1 *μ*M), garcinol was reported to stimulate intestinal cell growth, possibly due to production of hydrogen peroxide in the system, which may not be effectively quenched by the very low concentrations of garcinol [20]. Thus, the beneficial attributes of garcinol are dose dependent. This phenomenon of induction of cell growth at very low levels has been observed for other bioactive compounds as well including (−) epigallocatechin gallate [22].

Garcinol has also been shown to inhibit breast, prostrate and pancreatic cancer cell growth by Induction of apoptosis which was mediated by caspase-3 followed by downregulation of the NF*κ*B pathway [17, 30]. Chen et al., however, documented that garcinol may exert its anticarcinogenic effects in nicotine-induced breast cancer cells by induction of cell cycle arrest at the G0/G1 stage of mitosis [18]. In addition, this group showed that garcinol achieved inhibition of breast cancer cell proliferation by suppressing nicotine-induced nicotinic acetylcholine receptor (*α*9-nAChR) protein expression and downregulating type D cyclins associated with mitotic cell cycle arrest [18].

Tumor necrosis factor-related apoptosis-inducing ligand (TRAIL) is a cytokine known to elevate apoptosis via death receptors DR4 and DR5, via their interaction with Fas-associated domain (FADD), subsequently leading to activation of the caspase-3 pathway. The aforementioned cytochrome c/caspase 3 pathway can also be initiated by TRAIL by mitochondrial cleavage and capase 9 activation [16]. On treatment with garcinol, various cancer cell lines (colon, prostate, breast, kidney, leukemic, esophageal) exhibited increased apoptosis via an induction of death receptors DR4 and DR5 [16]. Thus its action does not appear to be specific to a particular cancer cell type. Moreover, garcinol was also reported to suppress the expression of prosurvival proteins survivin, bcl-2, XIAP, and cFLIP and conversely amplified proapoptotic pathways such as inducing cell membrane cleavage, increasing bax expression, and subsequent release of cytochrome c [16].

Platelet-derived growth factor receptors (PDGFRs) are implicated in medulloblastoma, the most common brain tumor of childhood. A recent report detailed the cytotoxic and antiapoptotic effect of garcinol against brain tumor cells (Daoy). The cytotoxicity, specifically against PDGFR-regulated Daoy cells, was accompanied with a significant S phase cell cycle arrest, downregulation of cyclin A and E, and activation of caspases [38].

### 4.3. Garcinol: Effect on Epigenetic Control

Alteration in genetic expression is yet another mechanism for the onset of cancer. Hokaiwado et al. investigated changes of gene expression in livers of rats treated with carcinogens and tumor promoters using a novel, three-dimensional microarray system, customized to meet experimental requirements. Using the customized microarray, garcinol was classified as chemoprotective against liver cancer at a concentration of 0.05% [24]. It was suggested that the likely mechanism of garcinol's anticarcinogenic activity involved the suppression of histone acetyltransferases [26]. Histone acetylation is a mode of chromatin remodeling, which enables gene transcription there by altering cell growth [26]. Histone acetylation and deacetylation is enabled by histone acetyltransferases (HATs) and histone deacetyltransferases (HDACs) [26]. Dysfunction of HAT can lead to cancers and therefore inhibitors of HAT may be of use in cancer therapeutics [22, 25, 26]. Garcinol inhibits HATs p300 and PCAF, thereby suppressing cell proliferation, which leads to cancer repression [26]. Interestingly, the specific inhibition of p300-HAT by a garcinol derivative, LTK-14, resulted in a noncytotoxic inhibition of HIV proliferation by preventing acetylation of HIV-infected cells [39].

Micro-RNAs (miR) are a family of highly conserved short RNA products (17–25 nucleotides) that control gene expression at the posttranscriptional levels regulating different cancer types. Although the functional roles of all miRs are not completely understood, they may act as oncogenes and/or tumor suppressors regulating cellular differentiation, proliferation, and apoptosis. We evaluated the effect of garcinol alone and in combination with gemcitabine on microRNA profile in human pancreatic cancer cell lines, BxPC-3 and Panc-1. MicroRNA microarray profile revealed a variation in expression of several microRNAs. Interestingly in our study, miR-21 (a signature of tumor aggressiveness) was significantly downregulated on treatment with garcinol as compared to gemcitabine or the combination treatment in gemcitabine-resistant cell line Panc-1, suggesting its role in acquisition of chemoresistance. (unpublished data).

### 4.4. Garcinol: Effect on Proliferation, Angiogenesis, and Metastasis

Angiogenesis and metastasis are hallmarks of advanced stages of carcinoma. Control at these stages is crucial for patient prognosis. Garcinol has shown very promising antiangiogenic and antimetastatic activities *in vitro*. Studies from our laboratory, demonstrate that garcinol downregulated MMP-9, IL-8, PGE-2, and VEGF, markers of angiogenesis and metastasis in pancreatic cancer cell lines, Panc 1 and BxPC3. The effects of garcinol were even more pronounced in the Panc1 cell line, carrying the *k-ras* mutation, implicated in the majority of human pancreatic cancer patients [31]. In another study, the effect of garcinol and other dietary phytochemicals on cell proliferation and migration was examined in rat liver bioassays and human hepatic cell lines. Cell invasion assays using Matrigel analysis showed a decrease in hepatocyte growth factor- induced cell invasion of HepG2 and MH1C1 cells by garcinol. However, the effects were not statistically significant and may need further investigation. According to recent study done on dysfunctional P53 cell line Hep 3B Garcinol has shown induction of ROS dependant apoptosis through death receptor, mitochondrial and modulating GADD153 [22, 23].

We have also explored the possible synergistic effects between garcinol and other phytochemicals, including curcumin as well as garcinol and currently available cancer therapeutics, including gemcitabine, in pancreatic cancer cell lines (unpublished data). In brief, using a combinatorial design, our results showed that garcinol and curcumin in concert show a greater decrease in cell growth via increased induction of apoptosis in pancreatic cancer cells, Panc 1 and BxPC3 than either agent alone.

In combination with the therapeutic, garcinol was seen to synergistically sensitize pancreatic cancer cells to gemcitabine *in vitro*. Human pancreatic cancer cell lines, BxPC-3 and Panc-1, harboring wild and mutant *K-ras *genotypes, respectively, were treated with garcinol and gemcitabine individually and in combination at different doses and times of incubation, to monitor growth inhibition and degree of apoptotic cell death. The combination of garcinol and gemcitabine showed a significant reduction in cell growth and increase in apoptosis as compared to respective individual treatments. Further, garcinol in synergism with gemcitabine induced its action by downregulating NF-*κ*B, VEGF, Il 8,* MMP-9 *and activating PARP cleavage. This further highlights the potential of garcinol in combinatorial therapeutic strategies.

Garcinol has also been shown to be involved in the genetic modification of Focal Adhesion Kinase (FAK), a key player in the regulation of major processes in the cell including cell proliferation, migration, and apoptosis [21]. Garcinol was shown to downregulate FAK activity by blocking its phosphorylation [21]. Subsequently, this inhibition was shown to alter the Bcl-2/BAX ratio, further leading to the activation of caspase-3 at a dose of 20 *μ*M [21].

## 5. Summary

Cancer, the uncontrolled growth and spread of abnormal cells, results from the accumulation of numerous sequential mutations and alterations in nuclear and cytoplasmic molecules [40]. Cancer progression is considered to involve three key steps: initiation, in which a normal cell is transformed into an initiated or abnormal cell, promotion, by which the initiated cell is converted into a preneoplastic cell, and progression, the process whereby the cells become neoplastic [41]. Cancer may be initiated due to multiple factors including exposure to carcinogens, repeated genetic damage by oxidative stress, chronic inflammation, or hormonal imbalance. This followed by a cascade of reactions, triggered by multiple signaling molecules makes it difficult to target a specific molecule responsible for the disease and thereby retard progression. Thus, to reduce cancer incidence and mortality rate and improve the survival time of cancer patients, new techniques and approaches must be developed to diagnose, prevent, and treat preinvasive lesions. Most chemotherapeutic treatments suffer from adverse toxic reactions leading to acute and delayed nausea, mouth ulcerations, fatigue, nerve damage, blood clots, anemia, and mild impairments [14]. Thus, it is important to develop effective preventative and/or therapeutic approaches either in the form of single agents or as combinations, which could potentially be both effective against cancer cell growth and relatively nontoxic. Recent evidence supports that nonnutritive components in diet have therapeutic benefits attributable to their pleiotropic effects including downregulation of survival signaling and simultaneous activation of multiple death pathways in cancer cells.

Here, we have attempted to present the potential preventative and/or therapeutic role of garcinol, the active component of *Garcinia* species against cancer progression. Although still preliminary in nature, recent evidence demonstrates that garcinol possesses multifunctional bioactivities including antimicrobial, anti-inflammatory, antioxidant, apoptotic, antitumorigenic, and perhaps anti-neurodegenerative as well.

Inflammation and oxidative stress are the key culprits implicated in numerous diseases, specifically in chronic conditions such as cancer, cardiovascular, and Alzheimer's disease. *In vitro* and some *in vivo* studies have shown that garcinol may inhibit these harmful chronic conditions from possibly manifesting and propagating via multiple mechanisms and sites of action. For instance, it behaves as a potent antioxidant with appreciable free radical scavenging activity, as also helping to avert inflammation, by suppressing proinflammatory signaling molecules, prostaglandins, leukotrienes and the signal transduction factor, NF-*κ*B.

Garcinol's inhibition of cancer growth of various types including pancreatic, prostate, breast, leukemia, colon, and its progression at different stages, is especially intriguing. Nonetheless, the full potential of this compound has yet to be elucidated. Research-based evidence pointing to the noncytotoxic nature against normal cells, combined with potent proapoptotic behavior against cancerous cells, and its antimicrobial effects may be a testament to the traditional use of the plant against multiple ailments. Forthcoming data on its synergistic effect with known drugs at subtherapeutic doses may also open up new avenues for efficient therapeutic regimens without or with minimal adverse side effects. In addition, to date, no toxic effects of Garcinol have been reported, even when given orally upto 0.05% in diet. However, further investigation to evaluate the range of its therapeutic potential is required. Most of the advances in the anticancer effects of garcinol, although mechanistically exciting, have been as a result of *in vitro* studies. These may or may not be entirely reflected in an *in vivo* animal model or a clinical situation. Thus the studies at present, although potentially very useful, call out for an immense need for carefully planned and executed studies in relevant animal models of various cancer types to confirm these findings.

## Figures and Tables

**Table 1:** List of studies investigating the role of Garcinol in different cancer types.

Type of cancer	Study type	Experimental details	Reference
Breast cancer	*In vitro*	MDA, MB231, MCF-7	[16]
	*In vitro*	MCF-7, MDA-MB231	[17]
	*In vitro*	Primary culture, MCF-7 MDA-MB231, AU 565 BT-483	[18]

Burkitt lymphoma	*In vitro*	TPA induced Ebvirus-EA activated raji cells	[19]

Colon cancer	*In vitro*	HT-29, HCT-116	[16]
	*In vitro*	HT-29, HCT-116, ICE-6	[20]
	*In vitro*	HT-29	[21]
	*In vivo*	Azoxymethane induced colonic aberrant crypt foci F344 rats	[15]

Esophageal cancer	*In vitro*	SEG-1	[16]

Hepatocellular carcinoma	*In vitro*	MH1C1-HEP G2	[22]
	*In vivo*	Hep3B	[23]
	*In vitro*	Carcinogen induced liver cancer F344 rats	[24]

HeLa cells	*In vitro*	HeLa core Histones-P300 HAT inhibition	[25]
	*In vitro*	HeLa Histones	[26]

Kidney cancer	*In vitro*	A293 (Human embryonic kinder carcinoma)	[16]

Leukemia	*In vitro*	KBM-5 (chronic Leukemia)	[16]
	*In vitro*	U937, K562, NB4, HL60	[27]
	*In vitro*	HL-60	[28]
	*In vitro*	HL-60	[15]

Lung cancer	*In vitro*	A-549	[29]

Medulloblastoma	*In vitro*	Daoy, growth factor,	[24]

Multiple myeloma	*In vitro*	U-266	[16]

Pancreatic cancer	*In vitro*	BxPC3	[30]
	*In vitro*	Panc-1, BxPC-3	[31]

Prostate cancer	*In vitro*	LNCaP, C4-2B, PC-3	[30]
	*In vitro*	PC-3	[16]

Tongue cancer	*In vivo*	4-nitroquinoline 1-oxide induced oral carcinogenesis in F344 rats	[32]
